# On the Validity of Detrended Fluctuation Analysis at Short Scales

**DOI:** 10.3390/e24010061

**Published:** 2021-12-29

**Authors:** Pedro Carpena, Manuel Gómez-Extremera, Pedro A. Bernaola-Galván

**Affiliations:** 1 Departamento de Física Aplicada II, E.T.S.I. de Telecomunicación, Universidad de Málaga, 29071 Malaga, Spain; mgomez_3@uma.es (M.G.-E.); rick@uma.es (P.A.B.-G.); 2 Instituto Carlos I de Física Teórica y Computacional, Universidad de Málaga, 29071 Malaga, Spain

**Keywords:** complex time series, power-law correlations, detrended fluctuation analysis, physiological time series

## Abstract

Detrended Fluctuation Analysis (DFA) has become a standard method to quantify the correlations and scaling properties of real-world complex time series. For a given scale *ℓ* of observation, DFA provides the function F(ℓ), which quantifies the fluctuations of the time series around the local trend, which is substracted (detrended). If the time series exhibits scaling properties, then $F\left(\ell \right)∼\ell^{\alpha }$ asymptotically, and the scaling exponent α is typically estimated as the slope of a linear fitting in the logF(ℓ) vs. log(ℓ) plot. In this way, α measures the strength of the correlations and characterizes the underlying dynamical system. However, in many cases, and especially in a physiological time series, the scaling behavior is different at short and long scales, resulting in logF(ℓ) vs. log(ℓ) plots with two different slopes, $\alpha_{1}$ at short scales and $\alpha_{2}$ at large scales of observation. These two exponents are usually associated with the existence of different mechanisms that work at distinct time scales acting on the underlying dynamical system. Here, however, and since the power-law behavior of F(ℓ) is asymptotic, we question the use of $\alpha_{1}$ to characterize the correlations at short scales. To this end, we show first that, even for artificial time series with perfect scaling, i.e., with a single exponent α valid for all scales, DFA provides an $\alpha_{1}$ value that systematically overestimates the true exponent α. In addition, second, when artificial time series with two different scaling exponents at short and large scales are considered, the $\alpha_{1}$ value provided by DFA not only can severely underestimate or overestimate the true short-scale exponent, but also depends on the value of the large scale exponent. This behavior should prevent the use of $\alpha_{1}$ to describe the scaling properties at short scales: if DFA is used in two time series with the same scaling behavior at short scales but very different scaling properties at large scales, very different values of $\alpha_{1}$ will be obtained, although the short scale properties are identical. These artifacts may lead to wrong interpretations when analyzing real-world time series: on the one hand, for time series with truly perfect scaling, the spurious value of $\alpha_{1}$ could lead to wrongly thinking that there exists some specific mechanism acting only at short time scales in the dynamical system. On the other hand, for time series with true different scaling at short and large scales, the incorrect $\alpha_{1}$ value would not characterize properly the short scale behavior of the dynamical system.

## 1. Introduction

Since a great diversity of real-world dynamical systems exhibit observable time series outputs characterized by scaling properties and complex correlations structure, many techniques have been developed in the last two decades to analyze this kind of time series and quantify adequately their properties, with Detrended Fluctuation Analysis (DFA) [1] being one of the most widely used, although other methods derived from fractal properties, complexity, and information theory are also common, such as Poincaré plots, fractal dimension, Hurst exponent (very related to DFA), different entropic techniques (Shannon, conditional, approximate, sample and multiscale entropies), and symbolic dynamics (see [2] for a recent review).

For a given scale *ℓ* of observation of the analyzed time series, DFA partitions the time series into windows of size *ℓ*, and quantifies the fluctuations of the time series within each window around the local trend. After averaging in all the windows of size *ℓ*, DFA provides the fluctuation function F(ℓ) which measures the average local fluctuations as a function of the observation scale (*ℓ*). In time series with perfect scaling and fractal power-law correlations, one finds asymptotically $F\left(\ell \right)∼\ell^{\alpha }$. The scaling exponent α is typically obtained as the slope of a linear fit of log(F(ℓ)) vs. log(ℓ). The exponent α quantifies the strength of the power-law correlations and characterizes the underlying dynamical system.

However, in many cases and especially when analyzing physiological time series associated with cardiac dynamics, the log(F(ℓ)) vs. log(ℓ) curve exhibits two different slopes (correlation behaviors) at short and large scales of observation. In this case, very often, *two* scaling exponents are obtained by fitting the log(F(ℓ)) vs. log(ℓ) at short ($\alpha_{1}$) and large ($\alpha_{2}$) scales [3,4,5], which quantify the short-term and long-term correlations, respectively. These two different scaling exponents are usually associated with the existence of different control mechanisms in the dynamical system which act at distinct time scales, so that $\alpha_{1}$ characterizes the control mechanism responsible for the short-time scales behavior, and, similarly, $\alpha_{2}$ is linked to the mechanism acting at long-time scales. The calculation of these two exponents has become a standard technique, when studying heart-rate variability [2,6], although it is also very common to distinguish between short-term and long-term scaling exponents in many other scientific fields. Some examples can be: the analysis of electroencephalograms for patients with Alzheimer’s disease [7], the behavior of glucose levels for patients with diabetes mellitus [8], the radon levels in soil associated with earthquakes [9], the stock market activity [10], the behavior of seismic signals [11]. or the properties of the trajectory of the center of pressure in the human postural control system [12].

In this work, we show that the use of $\alpha_{1}$ to characterize the correlations and scaling properties of dynamical systems at short time scales may lead to incorrect results. In particular, we show that, when analyzing artificial time series with *perfect* scaling, $\alpha_{1}$ systematically fails to detect the correct scaling at short scales. By using the Fourier Filtering Method algorithm (FFM), we are able to create synthetic time series with perfect scaling, i.e., characterized by a single scaling exponent α at *all* scales of observation. For these time series, F(ℓ) should behave as a perfect power-law at all scales (including short scales), $F\left(\ell \right)∼\ell^{\alpha }$. In this case, the log(F(ℓ)) vs. log(ℓ) plot should be a straight line with slope α, and therefore we should also obtain for short scales that $\alpha_{1}=\alpha$. However, we observe that, independently of the α value used to generate the FFM time series, the log(F(ℓ)) vs. log(ℓ) plot always exhibits a downwards curvature at short scales that has been reported previously [13] in time series with perfect scaling and some ad-hoc corrections to DFA were proposed, which has been attributed to overfitting in the detrended procedure [14]. The same effect is known to happen in the multifractal generalization of DFA [15]. However, we want to analyze systematically here how this phenomenon affects the determination of $\alpha_{1}$, since the curvature appears precisely in the range of scales where $\alpha_{1}$ is typically obtained, and the majority of authors do not consider the ad-hoc corrections proposed in [13]. The curvature produces a systematic overestimation of $\alpha_{1}$, which is in all cases larger than the correct exponent α, $\alpha_{1}>\alpha$.

We show that this overestimation is not due to effects produced by the finite time series length, but an intrinsic limitation of DFA, which only recovers the true scaling exponent α at larger scales of observation. In addition, the overestimation depends on the range of scales used to obtain $\alpha_{1}$, which varies considerably for different authors. The overestimation also depends on the value of the true scaling exponent of the time series.

In addition, we also analyze the behavior of $\alpha_{1}$ when studying time series with a scaling crossover separating two regions of true perfect scaling at both sides of the crossover, i.e., at short and large scales of observation. We create such time series by using a modified version of the Fourier Filtering Method, in which we can use as input the true values of $\alpha_{1}$ and $\alpha_{2}$ as well as the scale at which the crossover is located. In this case, F(ℓ) should exhibit two perfect linear behaviors in a log-log plot, with two different slopes for short and large scales and a transition between the two regimes around the scale of the crossover. As before, $\alpha_{1}$ is estimated as the slope of the linear fit of log(F(ℓ)) vs. log(ℓ) for short scales, and should coincide with the corresponding short-scale exponent used in the generation of the time series. However, we find that the estimated $\alpha_{1}$ value does not coincide with the true scaling exponent used to model the short scale behavior, which can be severely underestimated or overestimated. In this case, the estimated $\alpha_{1}$ value depends not only on the fitting range used to obtain it and of the real $\alpha_{1}$ value but, even worse, on the $\alpha_{2}$ value, i.e., time series generated with the same true $\alpha_{1}$ value and different true $\alpha_{2}$ values, provides different estimations of $\alpha_{1}$, although the short-term scaling properties are identical in all cases.

Therefore, the results we present here, obtained both for time series with perfect scaling and with scaling crossovers, suggest that, when analyzing real-world complex time series, the spurious value of the estimated $\alpha_{1}$ result could lead to incorrect interpretations of the short time behavior of the underlying dynamical system.

This paper is organized as follows: In Section 2, we introduce the connection of the autocorrelation function and Detrended Fluctuation Analysis, as well as how these two techniques should behave when applied to power-law correlated time series with perfect scaling. In addition, we also introduce how these time series can be generated by the Fourier Filtering Method. In Section 3, we introduce the question of the behavior of DFA at short scales, and how the short-term scaling exponent is usually determined. In Section 4, we present a systematic analysis of the behavior of DFA at short scales, and of the corresponding $\alpha_{1}$ exponent, when applied to time series with perfect scaling characterized by a single exponent. In this case, we show the overestimation effect described above, and systematically quantify it as a function of the true scaling exponent, and of the fitting range considered to estimate it. In Section 5, we introduce the generation of time series with two different perfect scaling behaviors for short and large scales, i.e., with known true values of $\alpha_{1}$ and $\alpha_{2}$, and study systematically the behavior of the estimated $\alpha_{1}$ as a function of the fitting range used to obtain it, and also as a function of the true $\alpha_{1}$ value and of the true $\alpha_{2}$ value. Finally, in Section 6, we present our conclusions.

## 2. Detrended Fluctuation Analysis and Autocorrelation Function in Time Series with Power–Law Correlations

In principle, the natural way of studying the correlations present in a time series for a given lag (*r*) is the determination of the autocorrelation function, C(r). For a stationary time series $\left\{x_{i}\right\}$ (i=1,2,…,N), the corresponding autocorrelation function can be calculated as

(1) $$C\left(r\right)=\frac{\langle x_{i}x_{i+r}\rangle -{\langle x_{i}\rangle }^{2}}{\langle x_{i}^{2}\rangle -{\langle x_{i}\rangle }^{2}}$$

where 〈…〉 denotes average over the whole time series. Without loss of generality, in the following, we assume that $\langle x_{i}\rangle =0$. When the time series $\left\{x_{i}\right\}$ is long-range power-law correlated, such as, for example, in fractional Gaussian noise (fGn), then its autocorrelation function C(r) behaves as a power law of the lag *r* [16]:

(2) $$C\left(r\right)≃\frac{H(2H-1)}{r^{2-2H}}∼\frac{\mathrm{sign}(1-\gamma )}{r^{\gamma }}$$

where *H* is the well-known Hurst exponent [17] with values in the range H∈(0,1), and then the autocorrelation exponent γ given by γ=2−2H, must be in the range γ∈(0,2). For H>0.5 (γ<1), the correlations are positive, while, for H<0.5(γ>1), the time series is anticorrelated. Note that, for the special case H=0.5(γ=1), the autocorrelation function vanishes, and the time series is uncorrelated (white noise behavior).

Similar power-law behavior for C(r) as that in (2) is obtained for time series generated using the *Fourier Filtering Method* (FFM) algorithm [18,19]. In this technique, a power spectrum of the type $S\left(f\right)∼1/f^{\beta }$ is imposed by creating a signal $\left\{X_{f}\right\}$ in the frequency *f* domain such that Re(Xf)=f−β/2cos(φf) and Im(Xf)=f−β/2sin(φf), where $\varphi_{f}$ is a random phase in the interval [0,2π). The time series $\left\{x_{i}\right\}$ obtained by Fourier transforming back $\left\{X_{f}\right\}$ presents by construction a power spectrum $S\left(f\right)∼1/f^{\beta }$. According to the Wiener–Khinchin theorem, the autocorrelation function of the final time series $\left\{x_{i}\right\}$ is $C\left(r\right)∼1/r^{1-\beta }$, and therefore the relation between the three exponents *H*, β and γ is [20]:

(3) $$H=1-\frac{\gamma }{2}=\frac{\beta +1}{2}$$

FFM has become the standard method to create a controlled power-law correlated time series, and it is used in many contexts for that purpose [21,22,23,24,25,26]

However, in many real-world time series, the autocorrelation function is not convenient to determine the exponent γ (or *H*), since C(r) is noisy and very sensitive to the time series size *N* [16,27], and it is only properly estimated for large *N*, very often not available in real experiments. This is the reason motivating the use of indirect methods to quantify correlations and scaling, such as Detrended Fluctuation Analysis (DFA), which is one of the most widely used.

Detrended Fluctuation Analysis was designed [1] to estimate the scaling properties of a given time series even in the presence of non-stationarities. DFA has been intensively tested and characterized by applying it to signals with different properties (trends, nonlinear filters, etc.) [28,29] and, since then, DFA has become one of the most standard methods used to analyze complex time series in many scientific fields. DFA works as follows: (i) Calculate the ’accumulated walk’ $Y_{j}$ of the analyzed time series $x_{i}$ of length *N*, such that

$$Y_{j}=\sum_{i=1}^{j}x_{i}.$$

(ii) Divide the walk $Y_{j}$ into boxes of equal length *ℓ* (the scale of observation). (iii) Within each box of length *ℓ*, calculate a linear fit of $Y_{j}$ to determine the *linear trend* within that box. The *Y* coordinate of the fitted line in each box is denoted by $Y_{\ell ,j}$. (iv) The walk $Y_{j}$ is detrended by subtracting the local trend $Y_{\ell ,j}$ in each box of length *ℓ*. (v) For a given box size *ℓ*, the root mean-square (r.m.s.) fluctuation function F(ℓ) for the detrended walk is calculated as:

(4) $$F\left(\ell \right)=\sqrt{\langle {\left(Y_{j}-Y_{\ell ,j}\right)}^{2}\rangle }$$

where, as usual, 〈…〉 means averaging over the whole time series. (vi) The above computation is repeated for a broad range of scales (box sizes *ℓ*) in order to provide a relationship between F(ℓ) and the scale *ℓ*. Scaling is present when

(5) $$F\left(\ell \right)∼\ell^{\alpha }$$

According to this last equation, when applying DFA to analyze real-world experimental data, the scaling exponent α is typically determined as the slope of a linear fit of log(F(ℓ)) vs. log(ℓ).

For stationary power-law correlated signals, α∈(0,1). The case α=0.5 corresponds to the absence of correlations (white noise), while α>0.5 indicates positive power-law correlations and α<0.5 corresponds to power-law anticorrelated time series. In this context, the DFA exponent α and the Hurst exponent *H* have the same value, H=α. In addition, DFA can be also applied to non-stationary long-range correlated signals of fractional Brownian motion type, and, in this case, 1<α<2. For example, for the standard Brownian motion, α=3/2.

In this work, we focus on stationary power-law correlated signals (0<α<1), where both the autocorrelation function and DFA can be applied. Note that, when the analyzed time series $\left\{x_{i}\right\}$ is stationary, an analytical relation between the autocorrelation function C(r) and the DFA fluctuation function F(ℓ) can be established. According to the derivation by Höll and Kantz [30], also obtained in a different manner by Talkner and Weber [31], for a time series with variance $\sigma^{2}$, we can write:

(6) $$F^{2}\left(\ell \right)=\sigma^{2}\left[(W\left(\ell \right)+\sum_{r=1}^{\ell -1}L\left(\ell ,r\right)C\left(r\right)\right]$$

with

(7) $$W\left(\ell \right)=\frac{\ell^{2}-4}{15\ell }$$

and

(8) $$L\left(\ell ,r\right)=\frac{1}{15(\ell^{4}-\ell^{2})}\left[(3r^{5}+\left(-20\ell^{2}+5\right)r^{3}+30\left(\ell^{3}-\ell \right)r^{2}+\left(-15\ell^{4}+35\ell^{2}-8\right)r+2\ell^{5}-10\ell^{3}+8\ell \right]$$

We want to remark that Equation (6) is an exact result, independently of the specific behavior of C(r) (positive, negative, power-law behaved or not, etc.). Therefore, Equation (6), which uses as input the values of C(r), provides an alternative way to the use of the standard DFA algorithm (4) to calculate the fluctuation function F(ℓ) for stationary time series. In the results presented in the next sections, when considering stationary time series, we have applied both techniques and have obtained identical results. In the case of non-stationary time series, we have used the standard DFA algorithm (4).

## 3. Detrended Fluctuation Analysis at Short Scales

As we stated in the Introduction, many real-world time series analyzed using DFA present a different scaling behavior at short and large scales *ℓ* of observation, i.e., the function F(ℓ) does not present a constant slope in the log(F(ℓ)) vs. log(ℓ) plot, but two different slopes at short and large scales. This change is usually attributed to the existence of two different mechanisms acting at different temporal scales which regulate the dynamics of the experimental system. For this reason, it is very common to characterize the analyzed time series, and the underlying dynamical system, by calculating two different scaling exponents, $\alpha_{1}$ and $\alpha_{2}$, obtained by a linear fitting of the log(F) vs. log(ℓ) at the two ends of the range of scales. In particular, $\alpha_{1}$ corresponds to the exponent obtained at short scales, and therefore $\alpha_{1}$ characterizes the short-term correlations. Many works, especially in physiology and more specifically in studies of heart rate variability, try to connect the $\alpha_{1}$ values with a diversity of healthy/pathological conditions. The range of scales considered for the $\alpha_{1}$ fitting depends on different authors: 3≤ℓ≤10 [32,33], 3≤ℓ≤11 [34,35], 4≤ℓ≤11 [36,37], 4≤ℓ≤12 [38,39] or even larger values (10≤ℓ≤30) [40]. More recently [41], it has been shown that the values of $\alpha_{1}$ evaluated in the interval 4≤ℓ≤16 seem to be a good biomarker of fatigue during extreme exercise.

Despite its usefulness in extracting information from physiological time series, we show here that, due to the intrinsic behavior of F(ℓ) at short scales, $\alpha_{1}$ has typically nothing to do with the actual scaling of the time series, i.e., we question the utility of $\alpha_{1}$ to characterize the short-term correlations in the way it is used in all the previous references, not its discriminating capacity in the classification of physiological time series.

In the next two sections, we systematically analyze the behavior of the estimated $\alpha_{1}$ in time series with a single scaling exponent (Section 4), and in time series with a scaling crossover and therefore two different scaling exponents at short and large scales.

## 4. Behavior of $\alpha_{1}$ in Time Series with Perfect Scaling

In this section, we show that, even for time series with perfect scaling behavior (i.e., with pure power-law fractal correlations), $\alpha_{1}$ does not provide an appropriate value characterizing such correlations. Here, we use the fitting range ℓ∈[3,12] for obtaining the results presented in this section, as a kind of consensus among the different ranges used in the bibliography described above. For completeness, some other values for the fitting range that are used in the bibliography will be also considered later.

**Figure 1:**
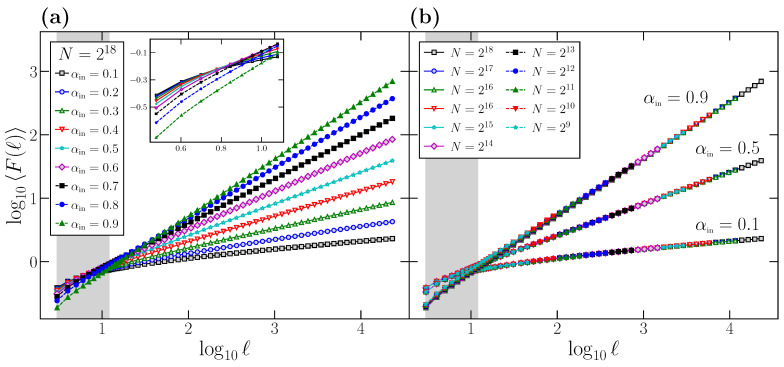
(**a**) The DFA average fluctuation function 〈F(ℓ)〉 obtained for FFM time series of length $N=2^{18}$ with different scaling exponents $\alpha_{\mathrm{in}}\in \left(0,1\right)$. The average is obtained by generating $10^{4}$ time series for each $\alpha_{\mathrm{in}}$ value. In the inset, we show a zoom of the region of short scales corresponding to the shaded rectangle, to better appreciate the curvature of the 〈F(ℓ)〉 functions in this region; (**b**) 〈F(ℓ)〉 obtained for three different $\alpha_{\mathrm{in}}$ values and for different time series length *N*. For any pair $\alpha_{\mathrm{in}}$ and *N*, we generate $10^{4}$ FFM time series to obtain the average 〈F(ℓ)〉. In both panels, the shaded rectangle corresponds to the range of scales usually considered to estimate the short-term scaling exponent $\alpha_{1}$.

**Figure 2:**
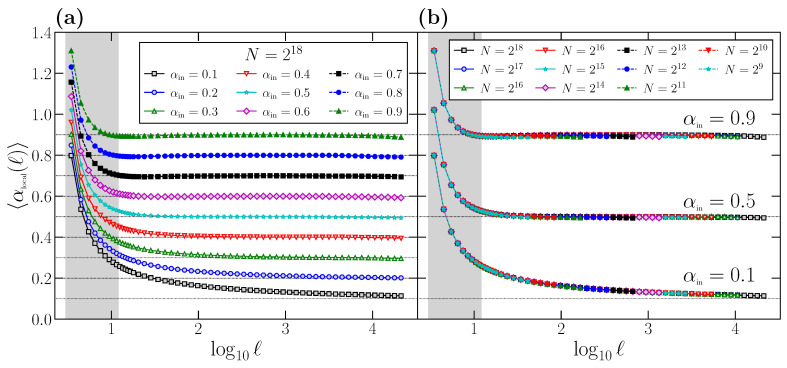
(**a**) Average local scaling exponent $\langle \alpha_{\mathrm{local}}\left(\ell \right)\rangle$ for the same set of time series used in Figure 1a with $N=2^{18}$. For each $\alpha_{\mathrm{in}}$ value, we generate $10^{4}$ time series, obtain for each one the curve $\alpha_{\mathrm{local}}\left(\ell \right)$ using Equation (9) and average the $10^{4}$ curves. (**b**) the same as in part (**a**), but for the time series used in Figure 1b for different *N*. In this case, we generate $10^{4}$ time series to obtain the average $\langle \alpha_{\mathrm{local}}\left(\ell \right)\rangle$ for each pair $\alpha_{\mathrm{in}}$ and *N*. The shaded area in both panels corresponds to the typical range of scales used to obtain $\alpha_{1}$.

**Figure 3:**
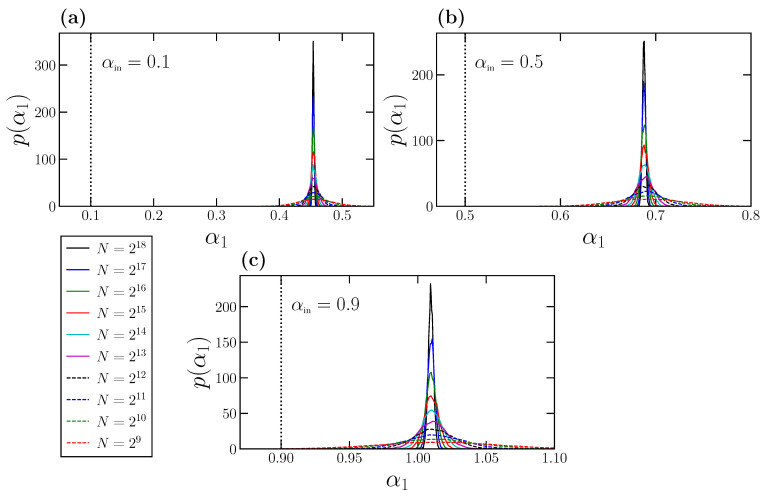
Probability densities $p(\alpha_{1})$ obtained for time series of different lengths *N* for three different values of $\alpha_{\mathrm{in}}$, 0.1 in (**a**), 0.5 in (**b**), and 0.9 in (**c**). For each pair *N*, $\alpha_{\mathrm{in}}$, we generate $10^{4}$ FFM time series and obtain the corresponding $10^{4}$$\alpha_{1}$ values, from where $p(\alpha_{1})$ is determined. In all panels, we also include as a vertical dashed line the true scaling exponent $\alpha_{\mathrm{in}}$ used to generate the corresponding FFM time series.

**Figure 4:**
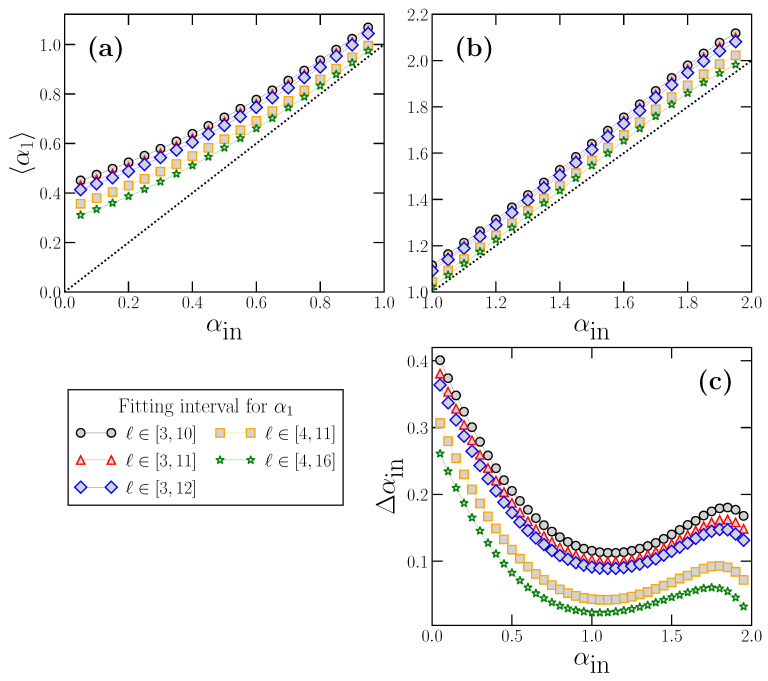
Behavior of $\langle \alpha_{1}\rangle$ as a function of the true scaling exponent $\alpha_{\mathrm{in}}$ for FFM power-law correlated stationary (**a**) and non-stationary (**b**) time series, using respectively values of $\alpha_{\mathrm{in}}$ in the range $0<\alpha_{\mathrm{in}}<1$ (**a**) and $1<\alpha_{\mathrm{in}}<2$ (**b**). In (**c**), we show the deviation $\Delta \alpha_{\mathrm{in}}$ as a function of $\alpha_{\mathrm{in}}$ in the whole range $0<\alpha_{\mathrm{in}}<2$. In addition to the $\alpha_{1}$ fitting interval [3, 12] that we have used in previous figures, we also show the results for other fitting ranges for $\alpha_{1}$, which are typically used in the bibliography.

We consider artificial time series generated using the Fourier Filtering Method described in Section 2. Due to the relation between the different scaling exponents (3) and as H=α for stationary power-law correlated time series, when we choose an input value $\alpha_{\mathrm{in}}$ and we create a time series with a perfect power-law behavior for the power spectrum, $S\left(f\right)∼1/f^{2\alpha_{\mathrm{in}}-1}$ (perfect scaling), we should obtain a perfect power-law behavior for the DFA fluctuation function F(ℓ) at all scales, $F\left(\ell \right)∼\ell^{\alpha_{\mathrm{in}}}$. However, we see below that this is not the case. In order to introduce the systematic errors when obtaining $\alpha_{1}$, in Figure 1, Figure 2 and Figure 3, we only present the results obtained for stationary time series of fractional Gaussian noise type, i.e., with a true scaling exponent in the range $0<\alpha_{\mathrm{in}}<1$. The behavior of $\alpha_{1}$ for time series of fractional Brownian motion type with $1<\alpha_{\mathrm{in}}<2$ exhibit similar properties, and it is not shown in Figure 1, Figure 2 and Figure 3 but will be included in Figure 4, where we show the final results for the systematic overestimation of $\alpha_{1}$.

In Figure 1a, we represent [42] the average behavior of the F(ℓ) function (〈F(ℓ)〉) for time series generated using the FFM algorithm with different $\alpha_{\mathrm{in}}$ values. For each $\alpha_{\mathrm{in}}$ value, we generate $10^{4}$ time series of length $N=2^{18}$ data points, calculate the F(ℓ) function for each one for scales *ℓ* in the range [3,N/10], and average the $10^{4}$F(ℓ) functions to obtain 〈F(ℓ)〉. We first observe how the behavior of 〈F(ℓ)〉 is correct for large scales, where all the curves in Figure 1a exhibit a slope in the log-log plot identical to the corresponding $\alpha_{\mathrm{in}}$ value.

However, if we observe the curves in Figure 1a, all of them present some degree of curvature at small scales, where the local slope deviates clearly from the correct $\alpha_{\mathrm{in}}$ value, which is only observed when the scale *ℓ* of observation increases. We remark that this curvature observed in the small *ℓ* region is not caused by a different behavior of the correlations at this scale, since all the time series considered have been generated to have a perfect power-law power spectrum, and therefore with the same scaling exponent at all scales. Indeed, the shaded area in Figure 1a corresponds to the range ℓ=[3,12]. i.e., the usual range where the scaling exponent $\alpha_{1}$ is obtained, and covers precisely the region where the curvature of the log(〈F(ℓ)〉) vs. log(ℓ) plots is more evident.

We also note that this curvature effect is not due to finite size effects. To show that this is the case, in Figure 1b, we choose as examples three different values of $\alpha_{\mathrm{in}}$ (although the results are general), and consider different time series length *N*. For each *N*, we generate $10^{4}$ FFM time series to obtain the corresponding 〈F(ℓ)〉 functions. We observe that, for the three $\alpha_{\mathrm{in}}$ values, the curves corresponding to different *N* overlap perfectly in the range [3,N/10], where DFA is calculated, and therefore the curvature observed at small scales is independent of the time series length *N*. This leads us to conclude that the curvature is a side effect of the DFA technique itself, which presents such curvature at small scales and only recovers the correct $\alpha_{\mathrm{in}}$ value in the large scale region.

This curvature effect can be better appreciated if we define the *local* scaling exponent $\alpha_{\mathrm{local}}\left(\ell \right)$ as the local slope of the log(F(ℓ)) vs. log(ℓ) curve:

(9) $$\alpha_{\mathrm{local}}\left(\ell \right)\equiv \frac{d\;\log(F(\ell ))}{d\;\log\ell }$$

For time series with perfect scaling, such as the ones generated with the FFM algorithm, we should obtain $\alpha_{\mathrm{local}}\left(\ell \right)=\alpha_{\mathrm{in}}$. However, due to the curvature of the F(ℓ) function, there is a clear deviation of $\alpha_{\mathrm{local}}\left(\ell \right)$ with respect to the correct value $\alpha_{\mathrm{in}}$ at short scales. This effect is shown in Figure 2a, where we plot the behavior of $\alpha_{\mathrm{local}}\left(\ell \right)$ for different $\alpha_{\mathrm{in}}$ values. All the curves have been obtained by generating $10^{4}$ time series of length $N=2^{18}$ for any value of $\alpha_{\mathrm{in}}$, obtaining for each one the corresponding function $\alpha_{\mathrm{local}}\left(\ell \right)$ using Equation (9), and averaging the results to get $\langle \alpha_{\mathrm{local}}\left(\ell \right)\rangle$. Again, the range of scales usually considered to determine $\alpha_{1}$ is shown as a shaded rectangle.

According to the results shown in Figure 2a, we can conclude that DFA provides the correct scaling exponent $\alpha_{\mathrm{in}}$ *asymptotically*: only for large or moderately large scales does the local slope $\alpha_{\mathrm{local}}\left(\ell \right)$ reach the true $\alpha_{\mathrm{in}}$ value, which is shown in all cases with a horizontal dashed line. However, at short scales, the local exponent $\alpha_{\mathrm{local}}\left(\ell \right)$ presents a large deviation with respect to the asymptotic value, specifically a clear overestimation since always $\alpha_{\mathrm{local}}\left(\ell \right)>\alpha_{\mathrm{in}}$. This deviation is larger for smaller $\alpha_{\mathrm{in}}$ values, especially for the anticorrelated cases $\alpha_{\mathrm{in}}<0.5$, but it is notorious in all cases. We remark that the scales where the deviation of $\alpha_{\mathrm{local}}\left(\ell \right)$ with respect to the correct scaling exponent $\alpha_{\mathrm{in}}$ is larger coincides with the shaded area, i.e., the range of scales used to determine $\alpha_{1}$.

Similarly to what we did in Figure 1, we proceed to show that the overestimation observed in $\alpha_{\mathrm{local}}\left(\ell \right)$ at short scales with respect to $\alpha_{\mathrm{in}}$ is not due to size effects: in Figure 2b, we show similar curves to the ones shown in Figure 2a, but obtained for a wide range of time series length *N*. We choose as examples the same three $\alpha_{\mathrm{in}}$ values considered in Figure 1b. For each combination of $\alpha_{\mathrm{in}}$ and *N*, we generate $10^{4}$ time series, determine for each one the corresponding $\alpha_{\mathrm{local}}\left(\ell \right)$ function in the range ℓ∈[3,N/10], and obtain the average of the $10^{4}$ curves. We observe that all the curves corresponding to the same $\alpha_{\mathrm{in}}$ value overlap perfectly on top of each other independently of *N*. Although shown only for three $\alpha_{\mathrm{in}}$ values, the behavior is completely general. Therefore, we can conclude that the deviation is not due to effects produced by the time series length *N*, but an intrinsic property of DFA, which systematically leads to a clear overestimation of $\alpha_{\mathrm{local}}\left(\ell \right)$ at short scales.

Since the short-term scaling exponent $\alpha_{1}$ is commonly estimated by the slope of a linear fitting of log(F(ℓ)) vs. log(ℓ) in the range ℓ∈[3,12], we observe from the results of Figure 1 and Figure 2 that, even for time series with perfect scaling, $\alpha_{1}$ will provide a spurious result not characterizing the correlations at those scales. Note that $\alpha_{\mathrm{local}}\left(\ell \right)>\alpha_{\mathrm{in}}$ for ℓ∈[3,12], and therefore $\alpha_{1}$, which is a kind of average of $\alpha_{\mathrm{local}}\left(\ell \right)$ in the fitting interval, will be also overestimated and will not properly represent the correlation properties at these scales.

Indeed, we can determine statistically the behavior of $\alpha_{1}$ for time series with perfect scaling. For that purpose, we choose different values of $\alpha_{\mathrm{in}}$, and for each one we consider a wide range of time series length *N*. For each combination of $\alpha_{\mathrm{in}}$ and *N*, we generate a $10^{4}$ time series with perfect scaling characterized by $\alpha_{\mathrm{in}}$ using the FFM algorithm. For each individual time series, we calculate the DFA fluctuation function F(ℓ) and obtain the corresponding $\alpha_{1}$ value by fitting log(F(ℓ)) vs. log(ℓ) for ℓ∈[3,12]. Therefore, we finally have $10^{4}$ individual $\alpha_{1}$ values for each pair $\alpha_{\mathrm{in}}$ and *N*, from where we can obtain numerically the corresponding probability density $p(\alpha_{1})$. In Figure 3, we show the results for the probability densities obtained for $\alpha_{\mathrm{in}}=0.1$ (panel a), 0.5 (panel b), and 0.9 (panel c). In each panel, we show the normalized probability densities for a wide range of time series length *N* values. In addition, we also show in each panel with a vertical dashed line the corresponding $\alpha_{\mathrm{in}}$ value, which truly characterizes the scaling and the correlations of the time series at *all* scales.

The behavior of $p(\alpha_{1})$ is quite similar in the three panels shown in Figure 3. Each individual density $p(\alpha_{1})$ exhibits a Gaussian-like shape with the peak centered at the corresponding mean value $\langle \alpha_{1}\rangle$. Interestingly, and since for a given $\alpha_{\mathrm{in}}$ all the $p(\alpha_{1})$ densities are centered in the same value independently of the time series length *N*, the expected $\langle \alpha_{1}\rangle$ value depends only on the corresponding $\alpha_{\mathrm{in}}$, but not on *N*. This property could have been anticipated by observing the overlapping of the curves shown in Figure 1b and Figure 2b for different *N* values. The effect of the time series length *N* is only reflected in the width of $p(\alpha_{1})$, which is larger for small *N* values, and decreases noticeably as *N* increases.

We observe in Figure 3 that the exponent $\alpha_{1}$ is systematically overestimated, and this effect can lead to spurious misinterpretations of the behavior of the analyzed time series, and therefore of the underlying dynamical system. For example, in Figure 3a, we analyze FFM time series fully characterized by $\alpha_{\mathrm{in}}=0.1$. This value indicates very strong power-law anticorrelations. However, the expected value $\langle \alpha_{1}\rangle$ is close to 0.5, corresponding to the absence of correlations (white noise behavior). In Figure 3b, we consider precisely $\alpha_{\mathrm{in}}=0.5$, and therefore the corresponding FFM time series are completely uncorrelated (white noises). However, we obtain in this case $\langle \alpha_{1}\rangle ≃0.7$ that would be interpreted as corresponding to positive and quite strong power-law correlations at short scales. In Figure 3c, we use $\alpha_{\mathrm{in}}=0.9$, so that the corresponding FFM time series are very strongly positively correlated. In this case, we obtain $\langle \alpha_{1}\rangle$ slightly larger than 1 that would be interpreted as corresponding to a non-stationary time series, for which α>1, although the FFM time series are stationary.

These overestimations of $\alpha_{1}$ could strongly affect the interpretation and implications of the results obtained with physiological time series. For example, Rogers et al. [41] show that the $\alpha_{1}$ value obtained from heart rate time series drops to 0.5 when runner’s fatigue increases. If we do not take into account these overestimations, we can conclude that fatigue makes the heart rate be random at short scales, whereas, in reality, the heart rate becomes highly anticorrelated at short scales.

These examples are useful to illustrate how $\alpha_{1}$ systematically overestimates the true scaling exponent $\alpha_{\mathrm{in}}$, and also that the overestimation depends on $\alpha_{\mathrm{in}}$ value. By repeating the same calculations presented in Figure 3 but for many $\alpha_{\mathrm{in}}$ values in the interval $\alpha_{\mathrm{in}}\in \left(0,2\right)$ (i.e., for stationary and non-stationary cases), we can obtain the dependence of the expected value $\langle \alpha_{1}\rangle$ on $\alpha_{\mathrm{in}}$, and quantify the overestimation $\Delta \alpha_{\mathrm{in}}$ defined as:

(10) $$\Delta \alpha_{\mathrm{in}}\equiv \langle \alpha_{1}\rangle -\alpha_{\mathrm{in}}$$

The results for $\langle \alpha_{1}\rangle$ as a function of $\alpha_{\mathrm{in}}$ are shown in Figure 4, where for clarity we have separated the results corresponding to stationary time series with $0<\alpha_{\mathrm{in}}<1$ (panel a), and to non-stationary time series with $1<\alpha_{\mathrm{in}}<2$ (panel b). In addition, we also include the dependence of $\Delta \alpha_{\mathrm{in}}$ on $\alpha_{\mathrm{in}}$ in panel c.

We observe how $\Delta \alpha_{\mathrm{in}}$ is larger for a stationary power-law strongly anticorrelated time series $\alpha_{\mathrm{in}}$ close to 0) and decreases as the true scaling exponent $\alpha_{\mathrm{in}}$ increases, reaching a minimum value around $\alpha_{\mathrm{in}}≃1$. After the minimum value, $\Delta \alpha_{\mathrm{in}}$ increases again in the non-stationary region and reaches a maximum at around $\alpha_{\mathrm{in}}≃1.8$ Although of variable extent, the overestimation always exists, and, as we have seen with the examples of Figure 3, this can lead to misinterpretations if the exponent $\alpha_{1}$ is considered to truly represent the short-term correlations of the analyzed time series.

We also include in Figure 4 the behavior of $\langle \alpha_{1}\rangle$ and $\Delta \alpha_{\mathrm{in}}$ as a function of $\alpha_{\mathrm{in}}$ for other values of the range of scales used to obtain $\alpha_{1}$ (typically used in the bibliography), in addition to the case ℓ∈[3,12], we have used in previous figures. We see that $\langle \alpha_{1}\rangle$ and $\Delta \alpha_{\mathrm{in}}$ depend also on the fitting interval considered, which is different for different authors, adding another degree of arbitrariness to the already difficult interpretation of the $\alpha_{1}$ value.

## 5. Behavior of $\alpha_{1}$ for Time Series with Scaling Crossovers

We consider in this section time series truly characterized by different short and long-term scaling exponents $\alpha_{1}$ and $\alpha_{2}$, and therefore with a scaling crossover at intermediate scales. These time series can be generated by using a modified version [43] of the Fourier Filtering Method described in Section 2. Essentially, the numerical procedure is identical to the standard FFM algorithm, but the power spectrum S(f), instead of as a single power-law, is modeled as:

(11) $$S\left(f\right)∼\left\{\begin{array}{ccc} \frac{1}{f^{2\alpha_{2}-1}} & \mathrm{if} & f\leq f_{c}\\ \frac{f_{c}^{2(\alpha_{1}-\alpha_{2})}}{f^{2\alpha_{1}-1}} & \mathrm{if} & f>f_{c} \end{array}\right.$$

**Figure 5:**
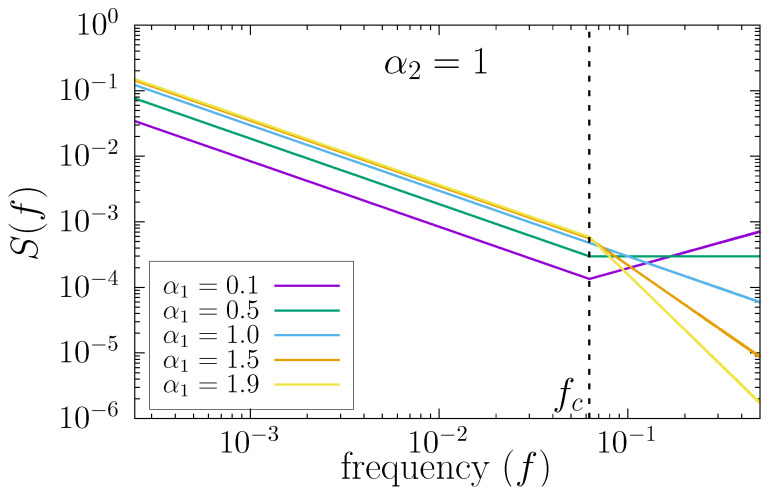
Examples of power spectra S(f) corresponding to Equation (11) with different DFA scaling exponents $\alpha_{1}$ at short scales (high frequencies) and $\alpha_{2}$ at large scales (low frequencies). The crossover occurs at frequency $f_{c}$, so that the scale of the crossover in the time domain is $\ell_{c}=1/f_{c}$. We have used a single numerical value $\alpha_{2}=1$, and several values for $\alpha_{1}$. In addition, we have considered $f_{c}=1/16$.

This equation corresponds to two different power-law behaviors of S(f) controlled by the exponents $\alpha_{1}$ (high frequencies) and $\alpha_{2}$ (low frequencies), with a crossover at frequency $f_{c}$. As an example, in Figure 5, we show the power spectrum of time series generated with this technique by using the numerical value $\alpha_{2}=1$ and different values of $\alpha_{1}$. The crossover frequency $f_{c}$ is indicated with a vertical dashed line, and we have used $f_{c}=1/16$ in the figure.

According to the definition of S(f) in Equation (11) and of the relation between the exponents of S(f) and DFA, when the corresponding signal in the frequency domain is Fourier-transformed back into time domain to obtain the time series $\left\{x_{i}\right\}$, the short scale behavior is truly characterized by a DFA exponent $\alpha_{1}$ and the large scale behavior, by a DFA exponent $\alpha_{2}$. The scale of the crossover, $\ell_{c}$, is given by $\ell_{c}=1/f_{c}$.

We note that this modified version of FFM has three input parameters, the scale of the crossover $\ell_{c}=1/f_{c}$ and the scaling exponents $\alpha_{1}$ and $\alpha_{2}$ that truly characterize the behavior of the final time series by construction. Since these exponents are inputs of the algorithm, from now on, we term them $\alpha_{1,\mathrm{in}}$ and $\alpha_{2,\mathrm{in}}$, respectively.

**Figure 6:**
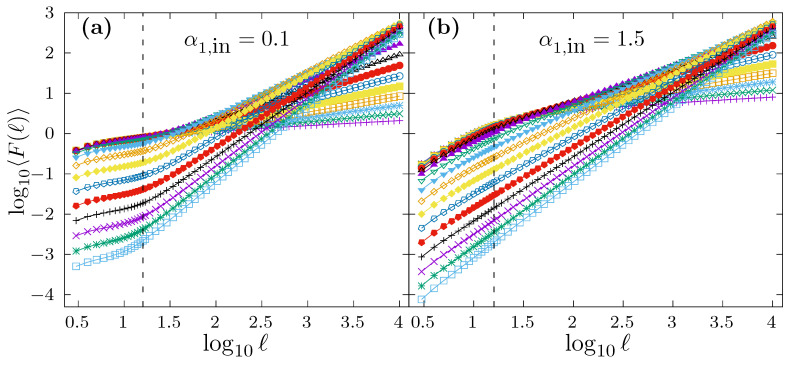
The DFA average fluctuation function 〈F(ℓ)〉 obtained for FFM time series of length $N=2^{18}$ with a scaling crossover at $\ell_{c}=16$, marked in both panels with a vertical dashed line. In (**a**), we fix $\alpha_{1,\mathrm{in}}=0.1$, and the different curves corresponds to $\alpha_{2,\mathrm{in}}=0.1,0.2,0.3,\ldots ,1.9$. In (**b**), we represent the same as in (**a**) but we fix $\alpha_{1,\mathrm{in}}=1.5$. In both panels, for any $\alpha_{2,\mathrm{in}}$ value, we generate $10^{4}$ time series to obtain the average 〈F(ℓ)〉.

In order to illustrate how DFA behaves when applied to time series with scaling crossovers generated by the modified FFM algorithm proposed in (11), in Figure 6, we show the average DFA fluctuation function 〈F(ℓ)〉 obtained for such kind of time series. In particular, we have considered in Figure 6 a scaling crossover at $\ell_{c}=16$ shown as a vertical dashed line in both panels. In panel a, we fix $\alpha_{1,\mathrm{in}}=0.1$, and each curve corresponds to $\alpha_{2,\mathrm{in}}$ values in the range 0.1,0.2,0.3,…,1.9. Figure 6b shows a similar case as in Figure 6a, but using a fixed value of $\alpha_{1,\mathrm{in}}=1.5$. In both panels, for each different $\alpha_{2,\mathrm{in}}$, we have generated $10^{4}$ time series of length $N=2^{18}$ to obtain the corresponding average curve 〈F(ℓ)〉. In all cases, we observe a change of slope in the log〈F(ℓ)〉 vs. log(ℓ) plot between short and large scales (as it should be).

**Figure 7:**
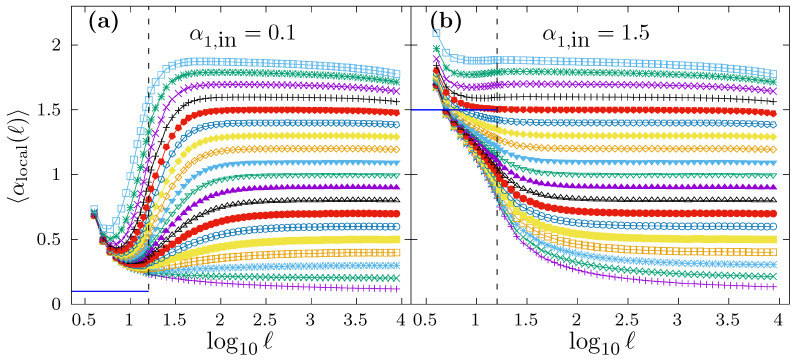
Average local scaling exponent $\langle \alpha_{\mathrm{local}}\left(\ell \right)\rangle$ for the same set of time series with $N=2^{18}$ and with scaling crossover used in Figure 6. In (**a**), we fix $\alpha_{1,\mathrm{in}}=0.1$, and the different curves corresponds to $\alpha_{2,\mathrm{in}}=0.1,0.2,0.3,\ldots ,1.9$. (**b**) shows the same as (**a**), but fixing $\alpha_{1,\mathrm{in}}=1.5$. The scaling crossover at $\ell_{c}=16$ is marked with a vertical dashed line. The true short-scale exponent $\alpha_{1,\mathrm{in}}$ is shown in each panel as a horizontal segment in the short-scale region. In both panels, for each value of $\alpha_{2,\mathrm{in}}$, we have generated $10^{4}$ time series to obtain the average $\langle \alpha_{\mathrm{local}}\left(\ell \right)\rangle$.

However, we want to investigate if the local scaling exponent in the short scale region for this kind of time series is able to recover the correct $\alpha_{1,\mathrm{in}}$ value. For that purpose, and for the same time series used to produce Figure 6, we show in Figure 7 the average local scaling exponent $\langle \alpha_{\mathrm{local}}\left(\ell \right)\rangle$ as a function of $\log_{10}\left(\ell \right)$. The scaling crossover at $\ell_{c}=16$ is shown in both panels as a vertical dashed line. In panel a, we consider the case $\alpha_{1,\mathrm{in}}=0.1$, while in panel b $\alpha_{1,\mathrm{in}}=1.5$. The different curves in both panels correspond to $\alpha_{2,\mathrm{in}}=0.1,0.2,0.3,\ldots ,1.9$, and the average $\langle \alpha_{\mathrm{local}}\left(\ell \right)\rangle$ is obtained by generating $10^{4}$ time series with $N=2^{18}$ for each $\alpha_{2,\mathrm{in}}$ value. In both panels, we indicate with a horizontal segment in the short scale region the true $\alpha_{1,\mathrm{in}}$ value used to generate the time series. In panel a, we observe how all the $\langle \alpha_{\mathrm{local}}\left(\ell \right)\rangle$ curves all well above the correct $\alpha_{1,\mathrm{in}}=0.1$ value. In this case, any fitting interval chosen in the short scale range will provide a drastic overestimation of $\alpha_{1,\mathrm{in}}$, although the specific estimation $\alpha_{1}$ value depends also on $\alpha_{2,\mathrm{in}}$. In panel b, we observe that some curves lie above the true $\alpha_{1,\mathrm{in}}$ value (approximately for $\alpha_{2,\mathrm{in}}>\alpha_{1,\mathrm{in}}$) while other curves lie below the true $\alpha_{1,\mathrm{in}}$ value (approximately for $\alpha_{2,\mathrm{in}}<\alpha_{1,\mathrm{in}}$). In the former case, the estimation $\alpha_{1}$ will overestimate the correct $\alpha_{1,\mathrm{in}}$ value, while, in the latter, $\alpha_{1,\mathrm{in}}$ will be underestimated. Either way, $\alpha_{1,\mathrm{in}}$ would not be properly determined in any case, and the particular estimated $\alpha_{1}$ value would depend on the true $\alpha_{2,\mathrm{in}}$, despite the fact that $\alpha_{1,\mathrm{in}}$ is identical in all cases.

**Figure 8:**
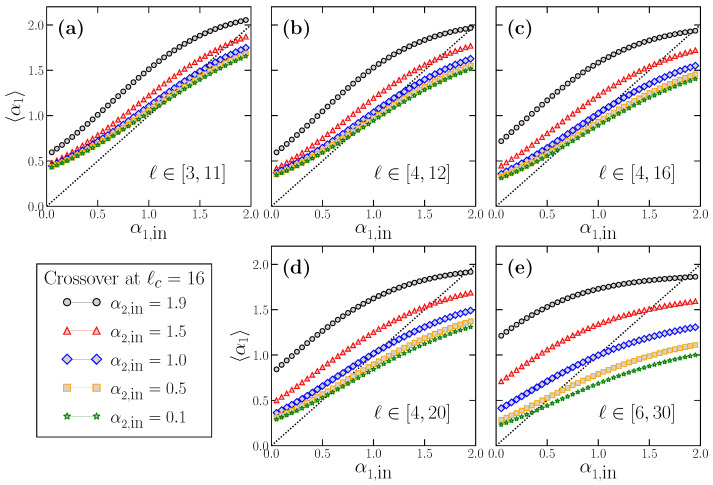
Behavior of the estimated $\langle \alpha_{1}\rangle$ as a function of the true short-term scaling exponent $\alpha_{1,\mathrm{in}}$ for different long-term scaling exponent $\alpha_{2,\mathrm{in}}$ values. For each combination of $\alpha_{1,\mathrm{in}}$ and $\alpha_{2,\mathrm{in}}$, we generate $10^{4}$ time series using the modified FFM algorithm with a crossover scale $\ell_{c}=16$, from where we obtain the average $\langle \alpha_{1}\rangle$. In each panel, we show the results for a different fitting range: [3,11] in panel (**a**), [4,12] in panel (**b**), [4,16] in panel (**c**), [4,20] in panel (**d**) and [6,30] in panel (**e**). The top panels correspond to fitting ranges below $\ell_{c}$, while, in the bottom panels, the upper limit of the fitting range is larger than $\ell_{c}$. In all cases, the dotted line in the diagonal corresponds to the perfect result $\langle \alpha_{1}\rangle =\alpha_{1,\mathrm{in}}$.

The examples shown in Figure 6 and Figure 7 indicate the practical impossibility of properly estimating the true $\alpha_{1,\mathrm{in}}$ for time series with scaling crossovers. Similarly to what we did in Section 3, we now proceed to analyze the behavior of the estimated short scale exponent $\langle \alpha_{1}\rangle$ for these time series. The results for $\langle \alpha_{1}\rangle$ as a function of the true $\alpha_{1,\mathrm{in}}$ are shown in Figure 8. We consider $\alpha_{1,\mathrm{in}}$ values in the range $\alpha_{1,\mathrm{in}}\in \left(0,2\right)$ to include stationary and non-stationary time series. Each panel corresponds to the use of a different fitting range to obtain $\alpha_{1}$, typically used in the bibliography. Within each panel, we present five different curves since we have considered five distinct values of $\alpha_{2,\mathrm{in}}$ to check its possible influence on $\langle \alpha_{1}\rangle$, which we know to exist according to Figure 7. For each combination of $\alpha_{1,\mathrm{in}}$ and $\alpha_{2,\mathrm{in}}$, we use the modified FFM algorithm in Equation (11) to generate $10^{4}$ time series with $N=2^{14}$ with the crossover scale at $\ell_{c}=16$. The $\alpha_{1}$ value for each individual time series is obtained by a fitting in the corresponding fitting range, and the resulting $10^{4}$ values of $\alpha_{1}$ are averaged to get $\langle \alpha_{1}\rangle$. In panels a–c, we consider fitting ranges for obtaining $\alpha_{1}$ below the crossover scale $\ell_{c}$, while in panels d and e, the upper limit of the fitting interval is above $\ell_{c}$. Note that, in a real-world time series with two different scaling behaviors at short and large scales, the true scale of the crossover is not exactly known a priori, so that situations such as the ones shown in panels d and e are realistic. In all cases, the dashed line in the diagonal of all panels corresponds to the line $\langle \alpha_{1}\rangle =\alpha_{1,\mathrm{in}}$, i.e., the expected behavior of $\langle \alpha_{1}\rangle$ in case of being correctly estimated.

**Figure 9:**
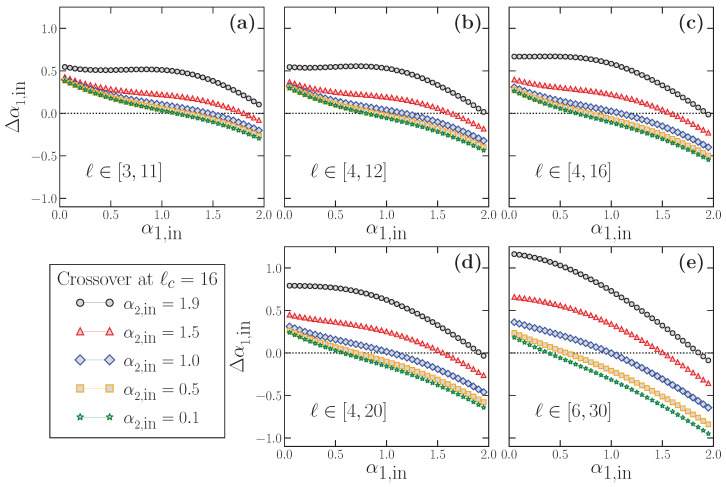
Deviation $\Delta \alpha_{1,\mathrm{in}}$ (Equation (12)) as a function of the true scaling exponent $\alpha_{1,\mathrm{in}}$. All the curves have been obtained from the data shown in Figure 6, so that each panel shows the results for a different fitting range: [3,11] in panel (**a**), [4,12] in panel (**b**), [4,16] in panel (**c**), [4,20] in panel (**d**) and [6,30] in panel (**e**). In all panels, the horizontal dotted line at $\Delta \alpha_{1,\mathrm{in}}=0$ corresponds to a perfect estimation of $\alpha_{1,\mathrm{in}}$.

Similarly, in Figure 9, we show the results for $\Delta \alpha_{1,\mathrm{in}}$ obtained from the data presented in Figure 8. In this case, the deviation of $\langle \alpha_{1}\rangle$ with respect to the true short-scale value is defined as:

(12) $$\Delta \alpha_{1,\mathrm{in}}\equiv \langle \alpha_{1}\rangle -\alpha_{1,\mathrm{in}}$$

We apply directly this last expression to the results shown in Figure 8 to obtain Figure 9. In all panels of this latter figure, the horizontal dotted line at $\Delta \alpha_{1,\mathrm{in}}=0$ corresponds to the perfect estimation of the true $\alpha_{1,\mathrm{in}}$.

The results shown in Figure 8 and Figure 9 have profound implications: First, $\langle \alpha_{1}\rangle$ practically never estimates properly the true $\alpha_{1,\mathrm{in}}$ value, as we suspected from the results of Figure 7. In this case, $\langle \alpha_{1}\rangle$ may overestimate ($\Delta \alpha_{1,\mathrm{in}}>0$) or underestimate ($\Delta \alpha_{1,\mathrm{in}}<0$), very often severely, the correct $\alpha_{1,\mathrm{in}}$. In general, for any fitting interval, we find that the overestimation happens for small $\alpha_{1,\mathrm{in}}$, and the underestimation for large $\alpha_{1,\mathrm{in}}$ values. In between these two extrema, and since $\langle \alpha_{1}\rangle$ and $\Delta \alpha_{1,\mathrm{in}}$ are smooth functions of $\alpha_{1,\mathrm{in}}$, there is an accidental single value of $\alpha_{1,\mathrm{in}}$ correctly estimated where the curves change from the over- to the underestimation region. However, this value is not robust since it depends on the fitting interval and the $\alpha_{2,\mathrm{in}}$ value considered.

The deviation $\Delta \alpha_{1,\mathrm{in}}$ depends obviously on the true $\alpha_{1,\mathrm{in}}$ value, but also on the fitting interval considered (see Figure 9), which is always quite arbitrary since the scaling of the crossover is not exactly known in real-world time series. We note that $|\Delta \alpha_{1,\mathrm{in}}|$ can have large values (i.e., strong under or overestimation of the true $\alpha_{1,\mathrm{in}}$) even when the fitting interval lies completely below the true crossover scale (Figure 8a–c and Figure 9a–c), and may worsen if the upper limit of the fitting interval is larger than the crossover scale (Figure 8d,e and Figure 9d,e).

However, in addition to these effects that preclude a correct estimation of the true $\alpha_{1,\mathrm{in}}$, there is another factor shown in Figure 8 and Figure 9 which questions severely the use of DFA at short scales, and that we already discussed partially when presenting Figure 7. We note that, for a fixed value of $\alpha_{1,\mathrm{in}}$, the corresponding estimated value $\langle \alpha_{1}\rangle$ (and therefore the deviation $\Delta \alpha_{1,\mathrm{in}}$) *depends also on the value of the true large-scale exponent* $\alpha_{2,\mathrm{in}}$. This effect implies a serious methodological problem: let us imagine two time series with exactly the same $\alpha_{1,\mathrm{in}}$ value (the same scaling behavior at short scales), but very different $\alpha_{2,\mathrm{in}}$ values. According to our results in Figure 8, if DFA is applied as usual at short scales to estimate the corresponding $\alpha_{1}$ value in these time series, the two estimated $\alpha_{1}$ values would be very different too, although the short-term scaling properties are identical in both time series, since they have the same true $\alpha_{1,\mathrm{in}}$. This case corresponds to imagining a vertical line for a fixed $\alpha_{1,\mathrm{in}}$ value at any of the panels of Figure 8. The line will cross the different curves at different $\langle \alpha_{1}\rangle$ values, which would be the estimated values provided by DFA, although, in all cases, the short-term scaling is the same. This effect corresponds exactly to the examples shown in Figure 7: while in all cases $\alpha_{1,\mathrm{in}}=0.1$ (panel a) or $\alpha_{1,\mathrm{in}}=1.5$ (panel b), a fitting in the short scale region (a kind of average of the corresponding $\langle \alpha_{\mathrm{local}}\left(\ell \right)\rangle$) would provide different estimated $\alpha_{1}$ values depending on $\alpha_{2,\mathrm{in}}$.

Similarly, the opposite situation is also possible: for two time series with different $\alpha_{1,\mathrm{in}}$ values, one can estimate the same $\alpha_{1}$ value applying DFA at short scales if the two time series have a different large-scale exponent $\alpha_{2,\mathrm{in}}$. This case corresponds to imagining a horizontal line at any fixed $\langle \alpha_{1}\rangle$ value in any of the panels of Figure 8. The line will cross the curves at very different true $\alpha_{1,\mathrm{in}}$ values, so that the time series truly have very different scaling properties at short scales but will be considered to have the same $\alpha_{1}$ value if DFA is used.

## 6. Discussion and Conclusions

In the last two decades, Detrended Fluctuation analysis has become a widely-used standard method to characterize the correlations and scaling properties of real-world complex time series. Within this context, many authors, especially in the field of physiology in the analysis of cardiac signals, study the scaling properties of the experimental time series by applying separately DFA at short and large scales of observation, therefore characterizing the time series by two exponents, $\alpha_{1}$ and $\alpha_{2}$, corresponding to short and large scales, respectively. If both exponents are different, and this happens very often, the difference is attributed to the existence of different mechanisms controlling the underlying dynamical system which act at different time scales, short and long range.

Here, we have shown that, when considering time series with perfect scaling, and therefore with a single exponent for all the scales of observation, DFA estimates correctly the real scaling exponent for large or moderately large scales of observation. However, if we calculate for these time series the exponent $\alpha_{1}$ in the range of short scales, we have observed a systematic deviation of $\alpha_{1}$ with respect to the correct and unique scaling exponent, which is in many cases largely overestimated by $\alpha_{1}$. This deviation depends not only on the value of the real scaling exponent, but also on the range of scales used to obtain $\alpha_{1}$. We have shown that this overestimation is not due to size effects of the time series, and therefore that it is an intrinsic property of DFA (artifact) at short scales.

In addition, when time series with a scaling crossover and two different scaling exponents at short and large scales are considered, the $\alpha_{1}$ value estimated by DFA can overestimate or underestimate (in many cases for a great amount) the correct short-scale exponent. The deviation of the estimated $\alpha_{1}$ with respect to the true exponent depends on the value of the true exponent itself, and of the fitting range considered (which varies among different authors) even if fitting ranges well below the scale of the crossover are considered. Even more importantly, the estimated value of $\alpha_{1}$ also depends on the value of the long-term scaling exponent, so that time series with identical short scale properties and different long scale properties will have different estimations of $\alpha_{1}$. This effect can also appear in the reverse way: we can find time series with the same estimated $\alpha_{1}$ values but with very different real short-term scaling properties if the long-term exponent is different.

We note that the results found in this work are of general applicability: note that the behavior of DFA at short scales we observe is due to the intrinsic mathematical properties of DFA, which only works properly asymptotically. i.e., for large scales. In the short scale region, the function F(ℓ) never behaves as a power-law, neither for signals with perfect scaling at all scales nor for signals with scaling crossovers, and this result is independent of the type of time series considered. Therefore, trying to fit F(ℓ) to a power-law in the short scale region always produces spurious results.

For all these reasons, the estimated value of $\alpha_{1}$ does not characterize properly the scaling properties and correlations at short scales, so that one has to be very careful when interpreting the meaning of $\alpha_{1}$ obtained for real-world experimental time series. On the one hand, if the experimental data truly exhibit perfect scaling with a single exponent, $\alpha_{1}$ will have a different value and could wrongly lead to the conclusion that there exists some specific mechanism acting on the dynamical system at short scales. On the other hand, for time series with two truly different scaling exponents produced by the characteristics of the dynamical system, the obtained $\alpha_{1}$ value will be also affected by the systematic deviation at short scales and will not coincide with the true short-scale exponent. In this latter case, the obtained $\alpha_{1}$ value will not characterize properly the real control mechanism acting at short scales on the dynamical system.
